# Abstracts presented at the meeting of the 23rd Congress of the Japanese Research Society of Clinical Anatomy

**DOI:** 10.1007/s00276-020-02559-8

**Published:** 2020-08-31

**Authors:** Keiichi Akita

**Affiliations:** Japanese Research Society of Clinical Anatomy (JRSCA), Tokyo, Japan

Introduction: Herewith are the abstracts of the presentations of the meeting of the 23rd Congress of the Japanese Research Society of Clinical Anatomy (JRSCA). The meeting was held at Tokyo Medical and Dental University, Tokyo, JAPAN on October 5, 2019.

## Plenary lecture 1

### Clinical anatomical study of the fasciae and ligaments in abdominal and pelvic cavities

#### Akita Keiichi

##### Department of Clinical Anatomy, Tokyo Medical and Dental University, Tokyo

The terms fasciae and ligaments are usually used in the anatomy of abdominal and pelvic cavities. However, these terms are often confused with the terms fasciae and ligaments, used in musculoskeletal anatomy. This confusion of terminology causes misunderstanding when defining the structures. The name “membrane” is also often misleading. It is important to determine whether the membrane is an epithelial membrane or a connective tissue membrane, as there is a significant difference in the structure of the two types.

I would like to organize the terms fascia and ligament that have been generally understood from previous anatomical studies of our group. Also, I would also like to try to specifically define the term membrane. The term fascia has been used in different ways in the musculoskeletal and abdominal/pelvic regions; the differences in these usages are clarified and discussed.

## Thoraco-abdominal regions

### Intramural distribution of the arteries in the stomach as demonstrated by X-ray examination with injected barium contrast medium

#### Eishi Haruka^1^, Yamaguchi Kumiko^1^, Hiramatsu Yoshihiro^2^, Akita Keiichi^1^

##### ^1^Department of Clinical Anatomy, Tokyo Medical and Dental University, Tokyo; ^2^Department of Radiology, Tachikawa Kitaguchi Kenshinkan, Tokyo

Introduction: On double-contrast views of the stomach, branching structures that appear as converging folds are frequently observed. However, in the endoscopic examination, there are no lesions in the same area. A previous study showed it could be that the “pseudo-converging folds” represent submucosal vessels. In this study, we show the distribution of intramural vessels throughout the stomach.

Materials and Methods: Twelve samples, including the stomach and the surrounding structures from 8% formalin-fixed cadavers were used for this study. The barium contrast medium was injected into the arteries surrounding the stomach. X-ray photography (Softex, Softex, JAPAN) was performed before and after the removal of vessels outside of the stomach. After examination by X-ray, three stomachs were examined with micro-CT analysis and histological analysis by EVG staining.

Results: By X-ray examination, arteries were vertical to the lesser and greater curvature side and had branches in various directions. In some cases, we observed folds that were parallel to the curvature, and these folds crossed arteries. Some of the arteries from the lesser and greater curvature sides anastomosed after insertion. Histological examination and micro-CT analysis revealed that most of the vessels lay in the submucosal layer. Most of the arteries in the submucosal layer were accompanied by veins.

Conclusion: Our findings of double-contrast examination suggested that vessels in the submucosal layer of the stomach wall could cause pseudo-converging folds. These results will help to differentiate false converging folds from true diseases and avoid unnecessary examinations.

### A case of hepatocellular carcinoma fed by the hepatic falciform artery originated from the right gastric artery

#### Matsumoto Junichi, Koda Wataru, Ogi Takahiro, Sugiura Takumi, Kobayashi Satoshi, Gabata Toshifumi

##### Department of Radiology, Kanazawa University Hospital, Kanazawa

A man in his 70 s with cirrhosis underwent S4 partial resection at the first occurrence of S4 hepatocellular carcinoma (HCC) was subsequently treated with transarterial chemoembolization (TACE) for recurrent lesions. This time, a new HCC appeared on the S3 liver surface, and TACE was planned again.

Before TACE, CT hepatic arteriography was performed to search for tumor feeders. The intrahepatic artery did not feed the tumor. Instead, a fine extrahepatic artery (we termed it a “fine artery”), originated from the proximal part of the right gastric artery (RGA), entered the hepatic falciform ligament just through the left caudal site of the paraumbilical vein at the umbilical portion of the left liver, and led to the tumor. Meanwhile, the left intrahepatic artery A4 branched as a hepatic falciform artery (HFA) running by the paraumbilical vein and by the fine artery mentioned above. Between the fine artery and the HFA, a fragile anastomosis was suspected inside the fat tissue at the liver surface.

In TACE, the angiogram via the RGA revealed the fine artery described above and the tumor staining which followed. We performed the embolization of the tumor via the artery, but not perfectly. The tumor was also visualized from the HFA branched from the A4, and we completed the additional embolization there in the same manner.

In a retrospective review of CT images, we found the occurrence of a communication between the RGA and the HFA during the follow-up before the HCC had developed. In other words, this vascular anastomosis was not congenital but rather acquired.

### Re-considering the hilar-mediastinal lymphatic system involved in lung cancer metastases: clinical Anatomical Study

#### Abe Miyuki^1,2^, Miura Masahiro^1^, Hamada Fumihiko^1^, sugio Kenji^2^

##### ^1^Department of Anatomy, Oita University Faculty of Medicine, Oita; ^2^ Department of Thoracic and Breast Surgery, Oita University Faculty of Medicine, Oita

Introduction: Systematic dissection from the superior to the inferior mediastinum is the standard procedure for lymph node (LN) dissection for primary lung cancer. Recently, the necessity of lobe-specific (selective) LN dissection has also been suggested from the viewpoint of minimally invasive surgery. However, the precise dissection of lobe-specific lymphatic pathways has not yet been fully elucidated.

Materials and Methods: Sixty-one patients with mediastinal LN metastases from primary lung cancer who underwent surgery at our institution were evaluated for the localization of metastatic LNs according to the lung lobe region. In addition, anatomical examination of the LN system was performed in extrapleural excised specimens of lungs and mediastinum from three adult cadavers.

Results: In patients with upper lobe lung cancer, no subcarinal LN metastases were found, and only paratracheal LN metastases were confirmed. In patients with lower lobe lung cancer, para-oesophagal LN and pulmonary ligament LN metastases were recognized only in the tumors with pleural infiltration.

In the anatomical examination, lymphatic vessels directly entered paratracheal LNs from the pleura of the upper lobe without passing through subcarinal LNs. The lymphatic vessels from the lower lobe all merged into the superior mediastinal LNs after passing through subcarinal LNs.

Conclusion: Subcarinal LNs may play a relaying role to the right and left, as well as upwards and downwards, in the mediastinum. However, the lymphatic flow from the upper lobe does not pass through this region, and attention should be paid to the flow route directly to paratracheal LNs.

### The assessment of the anatomical configuration of the thoracic duct at the jugulo-subclavian junction using magnetic resonance thoracic ductography

#### Okuda Itsuko^1^, Akita Keiichi^2^, Udagawa Harushi^3^

##### ^1^Department of Diagnostic Radiology, International University of Health and Welfare, Mita Hospital, Tokyo; ^2^Department of Clinical Anatomy, Tokyo Medical and Dental University, Tokyo; ^3^Department of Gastrointestinal Surgery, Toranomon Hospital, Tokyo

Purpose: Knowledge of the anatomical configuration of the thoracic duct (TD) is essential information to perform an intervention to treat the chylothorax. However, the detailed information of TD configuration at the jugulo-subclavian junction (JSJ) is not available to date. Thus, we assessed the TD configuration at the JSJ using magnetic resonance thoracic ductography (MRTD).

Materials and Methods: The institutional review boards (IRB) approved this study in our institutions. For the 80 subjects enrolled in this study, we performed MRTD on a 1.5-T MRI system without a contrast agent, and assessed the following three items: “the running course of the TD at JSJ”, “Incoming direction seen from the above at which TD flows into the venous angle after the thoracic outlet” and “minor variations”.

Results: The running courses of TD were classified into the following four types: sharp curve (33.7%); shallow curve (32.6%); horizontal course (18.0%); and ascending course (15.7%). The incoming direction at which TD flows into the venous angle was classified as follows: 5–6 o’clock (1.5%); 6–7 o’clock (9.2%); 7–8 o’clock (38.5%); 8–9 o’clock (38.5%); and 9–10 o’clock (9.2%); 10–11 o’clock (1.5%); 11–12 o’clock (1.5%). We found meandering (18.8%) and divergence patterns (11.3%) as minor variations.

Conclusion: Using MRTD, we succeeded in the non-invasive classification of the anatomical configuration of TD. This classification may be useful to perform the intervention of TD safely.

## Lunchtime seminar 1

### Anatomy of the deep branch of the superficial circumflex iliac artery

#### Yoshimatsu Hidehiko, Karasawa Ryo, Yano Tomoyuki

##### Cancer Institute Hospital, Plastic and Reconstructive Surgery

Background: The aim of this study was to describe the anatomical topology of the deep branch of the superficial circumflex iliac artery (SCIA) in fresh cadavers.

Methods: Twenty groin regions from 10 fresh cadavers were dissected. The characteristics and landmarks of the SCIA system, including branches to the sartorius muscle and the iliac bone, were examined. Perfusion of the sartorius muscle and the iliac bone via the deep branch of the SCIA was evaluated with indocyanine green angiography and computed tomography angiography.

Results: The superficial and deep branches were identifiable in all specimens. The deep branch in each case gave off branches to the sartorius muscle and the iliac bone. The cephalad portion of the sartorius muscle (up to 8 cm from the ASIS) and the superficial portion of the iliac bone (up to 2 cm from the iliac crest) were perfused by the deep branch of the SCIA.

Conclusion: In all specimens, both the superficial branch and the deep branch of the SCIA were found. The deep branch was found to consistently give off perfusing branches to the sartorius muscle and the iliac bone.

## Lunchtime seminar 2

### Profunda femoris artery perforator (PAP) flap: an anatomical study of the correlation of the perforators, the accessory saphenous vein, and the lymph collecting vessels and clinical applications of lymphatic vessels preserving the PAP (LpPAP) flap

#### Karakawa Ryo^1^, Yoshimatsu Hidehiko^1^, Yano Tomoyuki^1^, Chieh-Han John Tzou^2^

##### ^1^Cancer Institute Hospital, Plastic and Reconstructive Surgery; ^2^ Medical Faculty, Sigmund Freud University, Vienna, Austria

The profunda femoris artery perforator (PAP) flap is gaining popularity in microsurgical reconstruction. To establish a safer flap elevation technique, we focused on the two anatomical structures in the medial thigh area: the accessory saphenous vein and the lymph collecting vessels. The aim of this study was to describe the anatomical topology of these structures.

For the anatomical study, 19 posterior medial thigh regions from 10 fresh cadavers were dissected. We recorded the number, site of origin, the length, and the diameter of the pedicle. We also documented the course, the length, and the diameter of the accessory saphenous vein. From July 2018 to January 2019, 24 patients with soft tissue defects after tumor resection underwent reconstruction using PAP flaps. The lymph collecting vessels at the medial thigh area were identified using pre- and intra-operative ICG lymphography. A PAP flap was elevated taking care not to damage the lymph collecting vessels. After flap elevation, the anatomical correlation between the lymph collecting vessels and the anterior edge of the gracilis muscle was measured. The postoperative complications were assessed.

In all cadaveric specimens, the accessory saphenous vein could be found above the deep fascia. The average distance between the proximal thigh crease and the intersection of the accessory saphenous vein was 7.7 cm. PAP flaps survived completely in all clinical cases. The average length from the medial femoral epicondyle to the intersection of the collective lymphatic vessels and the anterior edge of the gracilis muscle was 9.7 ± 1.6 cm.

The anatomical study confirmed that the accessory saphenous vein did exist in all specimens and could easily be included in the PAP flap. The clinical study clarified a landmark point for the safer elevation of a skin paddle, which prevents postoperative lymphedema of the donor extremity.

## Pelvis

### Analysis of smooth muscle structure in the central region of male pelvic floor

#### Muro Satoru, Suriyut Junyaluk, Harada Masayo, Akita Keiichi

##### Department of Clinical Anatomy, Tokyo Medical and Dental University

Introduction: The pelvic floor support structure has been mainly considered for striated muscles and ligaments. In recent years, it has been found that smooth muscle tissue spreads on the pelvic floor. The aim of the present study was to clarify the spread of smooth muscle tissue on the male pelvic floor.

Materials and Methods: Two male cadavers were used for macroscopic examination. One male cadaver was used for histological analysis.

Results: In the superficial layer of the perineum, the muscle bundles of the bulbospongiosus, superficial transverse perineal muscle, and external anal sphincter surrounded the central region of the pelvic floor inferiorly and laterally. In the region surrounded by the striated muscle bundles, a cylindrical structure was observed. The cylindrical structure was composed of smooth muscle, and its superior part joined the longitudinal muscle of the rectum. In the deep layer of the perineum, a transverse muscle was observed between the levator ani and perineal membrane, connecting the bilateral ischiopubic rami. This muscle was composed of smooth muscle fibers extending anteriorly from the longitudinal and circular muscles of the rectum. In the deeper region, smooth muscle tissue was observed between the levator ani and anterior rectal wall. This tissue was composed of smooth muscle fibers extending from the longitudinal muscle.

Conclusion: In the central region of the male pelvic floor, smooth muscle tissue spread to fill the gap formed by the striated muscles and surrounding structures. Based on the findings, we considered the importance of smooth muscle in the male pelvic floor.

### Local anatomy of the anterior leaf of the vesicouterine ligament and vesicohypogastric fascia in radical hysterectomy

#### Nakamura Masaru^1^, Imanishi Nobuaki^2^, Aoki Daisuke^1^

##### ^1^Department of Obstetrics and Gynecology, Keio University School of Medicine; ^2^Department of Anatomy, Keio University School of Medicine

Introduction: We studied the local anatomy of the anterior leaf of the vesicouterine ligament (VUL) and vesicohypogastric fascia in a radical hysterectomy.

Materials and Methods: We observed the cervicovesical blood vessels and the connective tissue layer which continued from the umbilical artery and identified the origin of the cervicovesical blood vessels in radical hysterectomy. We also dissected a formalin-fixed female cadaver.

Results: After separation of the connective tissue of urinary bladder from the cervical fascia, we could discern the outline of the distal ureter near the ureterovesical junction. We gently separated the connective tissue of the so-called anterior leaf of the VUL enwrapping the ureter, and then the ureter with the connective tissue was completely rolled out laterally. We identified a cervicovesical vessel crossing over the ureter. We found that the cervicovesical vessel was a branch of the superior vesical artery. And, during cadaver dissection, we found that the connective tissue and the branches of the superior vesical artery were similarly observed.

Conclusion: We studied the precise anatomy of the connective tissue layer from the umbilical artery to the urinary bladder and the superior vesical artery. Our procedure based on the precise anatomy obtained in this study is reasonable anatomically as a method for separation of the vesicouterine ligament during radical hysterectomy.

### Ureterohypogastric nerve fascia/prehypogastric nerve fascia divided nerve branches to the bladder and uterus/vagina branches

#### Chikazawa Kenro^1,2^, Muro Satoru^2^ Akita Keiichi^2^, Imai Ken^1^, Tsutsumi Masahiro^2^ Yamaguchi Kumiko^2^, Kawata Tomoyuki^1^, KONNNO Ryo ^1^

##### ^1^Department of Obstetrics and Gynecology, Saitama Medical Center, The Jichi Medical University, Saitama; ^2^Department of Clinical Anatomy, Graduate School of Medical and Dental Sciences Tokyo Medical and Dental University, Tokyo

Introduction: Despite efforts for nerve-sparing radical hysterectomy to avoid bladder dysfunction, some patients have postoperative bladder dysfunction temporally or permanently. In surgery for rectum, ureterohypogastric nerve fascia (prehypogastric nerve fascia) is used as a landmark to avoid intraoperative injury to the dominant nerve of the internal anal sphincter urinary nerve, but its usefulness for gynecologic surgery is unknown. The purpose of the present study was to detect relationship of ureterohypogastric nerve fascia and nerve branches to the bladder, uterus, and vagina.

Materials and Methods: Five female cadavers were used in this study. The roots and branches of the pelvic plexus were dissected from the lateral surface. The branches of the pelvic plexus and nerves to the bladder, uterus, vagina were dissected. The positional relationships between the deep uterine vein, the vesical vein, the nerves, and the ureterohypogastric nerve fascia were carefully analyzed.

Results: The pelvic plexus was observed as a plate-like structure on the outside of the ureterohypogastric nerve fascia. In the nerve branch that started from the anterior upper corner of the pelvic plexus, anterior branches and anteromedial branches were found. Anterior branches located lateral to the ureterohypogastric nerve fascia were distributed to the bladder.

Anteromedial branches and deep uterine vein penetrated the ureterohypogastric nerve fascia, and then distributed to the uterus and vagina.

Discussion: We perform radical hysterectomy using this knowledge. In the surgical procedure, we preserve the ureterohypogastric nerve fascia to place the nerves on the lateral side of the uterus. It is only for cervical cancer under 2 cm tumor diameter that we perform this nerve-sparing radical hysterectomy. We performed this surgery for 3 patients, and they have a negative surgical margin and negative parametrial invasion. Also, both patients achieved catheter removal on postoperative day 3 and a postvoid residual urine volume of less than 50 ml by postoperative day 3. Both micturition and bladder fullness were good. The ureterohypogastric nerve fascia separated nerve branches to the bladder and uterus/vagina branches.

### Observation of the fine structure of fascia through the latest endoscope: changing the concept of fascia

#### Kawashima Kiyotaka

##### Tochigi Cancer Center Hospital

Introduction: In surgery, the layer structure of fascia has been thought to be a good landmark for surgeons for dissection. Recently the surgical anatomy, and in particular, the layer structure of fascia around the prostate has become precisely defined. However, discordance between surgeons still remains regarding the recognition of fascial layers. Recently fascial research has greatly progressed. Some fascia researchers describe fascia as a three-dimensional web-like structure mainly consisting of collagen fibers and elastin fibers. Dr. Jean-Claude Guimberteau, a French plastic surgeon, observed dynamic movement of fibers of fascia in the human living body during surgery using the latest high-resolution endoscope. Fascia researchers think fascia is not a film-like structure that merely covers and connects organs but rather it is a three-dimensional web-like structure that makes, maintains and protects the shape of the body against outer force and integrates and regulates the entire body. Some fascia researchers use the term “fascia system”.

Purpose and Methods: We observed the fine structure of fascia by high-resolution endoscope during radical prostatectomy for prostate cancer with a minimal incision to verify the nature of the structure of fascia.

Results and Discussion: In a highly magnified view, red blood cells floating in the capillary and the movement of lymph vessels were observed. It means we could observe up to 10 μm. In this high magnification, crossing of fibers is observed in fascia. By traction, fascia appeared as a three-dimensional network-like structure and the construction of fibers changed dynamically depending upon the force. Fascia showed various phenotypes. It was thought that we should recognize fascia to be connective tissue that changes from loose to dense seamlessly depending upon purpose or needs, such as strength and movement. We thought that the term of fascia should be used in a broader meaning.

## Head and neck regions and back regions

### Analysis of the attachment of the esophagus on the anterior wall

#### Miwa Koh^1^, Tsutsumi Masahiro^1^, FUKINO Keiko^2^, Akita Keiichi^1^

##### ^1^Department of Clinical Anatomy, Tokyo Medical and Dental University, Tokyo; ^2^Department of Orofacial Development and Function, Tokyo Medical and Dental University, Tokyo

Introduction: The upper esophageal closing occurs in the transitional region between the hypopharynx and the upper esophagus. Although numerous reports have discussed the anatomical structure of the posterior wall of the transitional region, few studies have focused on its anterior wall. The aim of this study was to investigate the contribution of the anterior wall in upper esophageal closing.

Materials and Methods: A total of 13 Japanese cadavers were evaluated in this study. Six and seven were used for macroscopic and histological analysis, respectively.

Results: The anterior wall of the transitional region consisted of three muscular layers: the outer longitudinal layer, the inner circular layer, and the longitudinal muscle bundles located on the inner surface of the inner circular layer. Each muscle was continuous with the tendinous structure, which attached to the upper region of the cricoid cartilage. The outer longitudinal layer was thick on the upper anterolateral wall and continuous with the tendinous structure at the lower border of the cricoid cartilage. In addition, the outer longitudinal layer was thin on the anteromedian wall and some of the inner circular layers were exposed to the outer surface. Both the inner circular layer and the longitudinal muscle bundles were continuous with the inner tendinous structure. The inner circular layer above the lower border of the cricoid cartilage was characterized by its oblique running direction. Histological analysis showed that some of the tendinous structure extended to the aponeurosis of the transverse arytenoid.

Conclusion: The anterior wall of the transitional region between the hypopharynx and the upper esophagus might contribute to the upper esophageal closing by the thickness of the anterolateral wall of the outer longitudinal layer and the oblique running direction of the inner circular layer.

### Spatial distribution of the palatopharyngeus in consideration of the opening of the orifice of the oesophagus

#### Fukino Keiko^1^, Tsutsumi Masahiro^2^, Nimura Akimoto^3^, Miwa Koh^2^, Ono Takashi^1^, Akita Keiichi^2^

##### ^1^Department of Orthodontic Science, Tokyo Medical and Dental University, Tokyo; ^2^Department of Clinical Anatomy, Tokyo Medical and Dental University, Tokyo; ^3^Department of Functional Joint Anatomy, Tokyo Medical and Dental University, Tokyo

Introduction: For the swallowing, the velopharyngeal closure, elevating the larynx and opening the oesophagus are important movements. We reported that the palatopharyngeus contributes to the velopharyngeal closure and elevating the larynx, according to the directions of the muscle fibers and attachment sites of the palatopharyngeus. Reports suggest that the palatopharyngeus might continue to the muscle bundle of the oesophagus, as well as to the inferior pharyngeal constrictor. However, the relationship between the palatopharyngeus and the inferior constrictor remains unclear. In the present study, we examined the attachments of the palatopharyngeus to the inferior constrictor.

Materials and Methods: We evaluated 10 halves of 5 heads from Japanese cadavers (one male and 4 females; average age, 83.9 years). Of the 10 halves, nine halves of four heads were assigned to macroscopic examinations and one head half to histological examinations.

Results: A part of the palatopharyngeus originated from the superior surface of the soft palate, ran inferoposteriorly and attached to the pharyngeal raphe. A part of the palatopharyngeus originated from the inferior surface of the soft palate, ran anteroinferiorly and attached to the thyroid cartilage, epiglottis and inner surface of the inferior constrictor. In the histological examinations, connective tissue fibers including the elastic fibers around the muscle fibers continuous to the palatopharyngeus ran between the muscle fibers of the inferior constrictor.

Conclusion: When the palatopharyngeus constricts, the inferior constrictor is elevated anterolaterally and helps to open the orifice of the oesophagus. Therefore, the palatopharyngeus might have the role of opening the orifice of the oesophagus.

### A novel dissection method for the facial soft tissue of a formalin-preserved cadaver

#### Watanabe Koichi^1^, Hayakawa Kouji^2^, Iwanaga Joe^1,3^, Tabira Yoko^1^, Saga Tsuyoshi^1^, Inoue Eiko^1^, Yamaki Koh-ichi^1^

##### ^1^Division of Clinical and Gross Anatomy, Department of Anatomy Kurume University School of Medicine, Fukuoka; ^2^ Hakusan Clinic, Oita; ^3^Seattle Science Foundation, WA, USA

Purpose: As formalin-preserved cadavers are widely used, it is relatively easy to obtain the tissues. However, due to the hardening of the tissue, it is considered that the tissue is not suitable for facial soft tissue that requires delicate dissection. The purpose of this study was to develop a method to unveil the fibrous tissue structures including SMAS and to retain ligaments using formalin-fixed specimens.

Materials and Methods: For this study, 10 sides of hemifacial tissue were used. The extracted tissue was cut at a width of 1 cm to create cross-sections. After pinning and fixing the bone on the wooden plate, the skin was pulled outward with strings to overextend the soft tissue, and the adipose tissue was removed under an operating microscope to dissect the fibrous tissues.

Results: In all cases, it was easy to observe the fibrous structure after the removal of the fat. In particular, it was easy to dissect and observe the three-dimensional structure of the SMAS and various retaining ligaments in each section. Furthermore, differences in the density of the fibers depending on each fat compartment could be observed by dissection of adipose tissue.

Conclusion: This method has a great advantage to observe the structure of the tissue three-dimensionally. A cut specimen using this method has depth so that three-dimensional structures can be observed. The layers and spaces in the soft tissue become clear by pulling the skin outward.

### Anatomical characteristics of the serratus anterior muscle branch, which is sacrificed for pedicled latissimus dorsi muscle flap transfer: a clinical anatomical study

#### Miura Masahiro^1^, Abe Miyuki^1,2^

##### ^1^Department of Anatomy, Oita University Faculty of Medicine, Oita; ^2^ Department of Thoracic and Breast Surgery, Oita University Faculty of Medicine, Oita

Introduction: In this study, in order to ascertain the effectiveness of severing the muscle branch that originates from the thoracodorsal artery (TDA) in sacrificing it for pedicled latissimus dorsi muscle transfer (PLDMT), the anatomical characteristics of the arteries supplying the serratus anterior muscle (SA) were investigated.

Materials and Methods: Fifty-six sides of 28 Japanese cadavers were carefully studied and analyzed relative to the TDA-originating SA branch and the SA distribution pattern of the lateral thoracic artery (LTA). Changes in the route of the axillary artery, which affect the morphology of the origin of the subscapular artery (SSA), were investigated based on the relationship between the SA branch and the route pattern.

Results: The SA branches were classified as follows: (i) branches from the standard SSA (64%), and (ii) branches from the subscapular superficial artery (36%). Both types of SA branches ran together with the long thoracic nerve. In contrast, in the upper bundle of the SA, the muscle branches originated from the descending scapular artery and suprascapular artery.

Conclusion: The SA branch is a feeding branch exclusively for the lower SA bundle. The severing of this branch was therefore conjectured to be a potential cause of postoperative SA atrophy and/or ischemia inside the sheath of the long thoracic nerve. The SA branch that is usually presented was severed, but the LTA provides a substitute supply; however, in developmental terms, the LTA originates from the intercostal arteries and is therefore considered an insufficient substitute for this artery.

### The topology of the superior gluteal region with special reference to the course of the superior cluneal nerves and the composition of the thoracolumbar fascia

#### ishikawa Hirotaka^1,2^, Sakuraya Tohma^1^, Emura Kenji^3^, Arakawa Takamitsu^1^

##### ^1^Kobe University Graduate School of Health Sciences, Hyogo; ^2^Let’s Rehabili Elderly Day Care Kakegawa, Shizuoka; ^3^Faculty of Health Care Sciences, Himeji Dokkyo University, Hyogo

Introduction: Entrapment neuropathy of the superior cluneal nerves has been proposed as the cause of the low-back pain because these nerves pass through the posterior layer of the thoracolumbar fascia near the iliac crest and the symptoms are varied. In previous research, anatomical variations of the courses of the superior cluneal nerves and the composition of the thoracolumbar fascia have been reported, and therefore, topology of superior gluteal region is important to discuss entrapment neuropathy of the superior cluneal nerves.

Materials and Methods: The superior cluneal nerves were dissected near the iliac crest on both sides of one cadaver. Constitution of the superior cluneal nerves and the thoracolumbar fascia were recorded by line drawings and digital photographs.

Results: On the right side, five superior cluneal nerves appeared. The most lateral branch pierced the aponeurosis of the internal oblique. The others pierced the upward continuation of the aponeurosis of the gluteus medius. The superior cluneal nerves spread within the subcutaneous tissue from the proximity of the posterior superior iliac spine to the level of the greater trochanter of the femur. On the left side, three superior cluneal nerves appeared. All the branches pierced the aponeurosis of the internal oblique. Left superior cluneal nerves extended the subcutaneous tissue from the inferior margin of the iliac crest to the level of the greater trochanter of the femur.

Conclusion: These results suggest that the symptom of the low-back pain might differ inside when entrapment neuropathy of the superior cluneal nerves occurs.

## Locomotor system

### Anatomical variation of relationship between the subclavius muscle and coracoclavicular ligament: its clinical importance

#### Izumida Mizuki^1^, Emura Kenji^2^, Sakuraya Tohma^3^, Yamamoto Rintaro^1^, Ikezawa Hideki^3,4^, Shimizu Takahiro^5^, Arakawa Takamitsu^3^

##### ^1^Kobe University School of Medicine Faculty Health Science, Hyogo; ^2^ Himeji Dokkyo University Faculty of Health Care Sciences, Hyogo; ^3^ Kobe University Graduate School of Health Science, Hyogo; ^4^ Kiba Hospital, Hyogo; ^5^ Kobe University Graduate School of Medicine

Introduction: When clavicle fractures or dislocation of the acromioclavicular joint occur, the coracoclavicular ligament is possibly injured. The subclavius muscle usually inserts near the attachment site of the coracoclavicular ligament, and therefore, this muscle has the possibility to be impaired by injuries of the coracoclavicular ligament. In the present study, the topology of the insertion of the subclavius muscle and attachment of the coracoclavicular ligament was examined.

Materials and Methods: The relationship of the subclavius muscle and coracoclavicular ligament was analyzed in 46 sides of 29 Japanese cadavers that had been supplied to the anatomical course of Kobe University School of Medicine. Constitutions of the muscle bundle of the subclavius were examined in 10 sides.

Results: All subclavius muscles arose from first rib and costal cartilage and its muscle bundles were separated into two, superomedial and inferolateral parts. The superomedial part was fusiform muscle, ran in the superolateral direction, then inserted around the conoid tubercle of the clavicle. The inferolateral part was bipennate muscle with a greater muscle bundle than the superomedial part and this part covered the entire superomedial part from inferior aspect. Medial bundles of the inferolateral part inserted into the tendon of the superomedial part, while the insertion of the remaining lateral bundles varied. Type 1 of this muscle inserted into an area apart from the conoid ligament (26.1%). Type 2 of this muscle tendon fused with the conoid ligament (58.7%). Type 3 inserted into the conoid ligament (15.2%).

Conclusion: The subclavius muscle might be injured in conoid ligament injury at a high rate (73.8%; Types 2 and 3).

### An anatomic study regarding fibrous structures attached to the ulnar corner of the radius: relationship with volar rim fracture

#### Saka Natsumi^1,2^, Nimura Akimoto^3^, AKITA Keiichi^1^

##### ^1^Department of Clinical Anatomy, Graduate School of Medical and Dental Science, Tokyo Medical and Dental University, Tokyo; ^2^Department of Orthopaedics, Teikyo University School of Medicine, Tokyo; ^3^Department of Functional Joint Anatomy, Tokyo Medical and Dental University, Tokyo

Introduction: The current study investigated the anatomical features of the distal radius, particularly the volar ulnar corner, to determine a clear understanding of the attachment of the disc proper and the joint capsule.

Materials and Methods: We investigated the morphological features of the distal radius of 10 cadaveric wrists using micro-computed tomography (micro-CT). We macroscopically analyzed five specimens to examine the soft tissue attachment of the volar ulnar corner of the distal radius. We also histologically analyzed 1 specimen in the sagittal plane.

Results: The distal radius had a prominent area at the volar ulnar corner. Macroscopic observation showed that the disc proper and the volar capsule attached to this prominence. By histological analysis, the deep layer of the volar capsule was observed as thick oblique fibers between lunate and radius. These fibers attached to the radius from the articular cartilage to the distal bony demarcation point of the radius. The middle layer of the capsule was observed as transverse fibers. At the most volar side, the superficial layer of the volar capsule was observed as loose connective tissue.

Conclusion: The disc proper and the volar capsule attached to the volar ulnar corner of the radius in complex fashion and they support the radio-lunate stability as well as the stability of the distal radioulnar joint. In relation to volar rim fracture, not only the force in the sagittal direction but also the avulsion and rotational force to the ulnar side might explain the cause of this fracture.

### Morphological structure of the Palmar Carpal Ligament, and the relationship between this ligament and the palmar branch of the ulnar nerve

#### Tabira Yoko^1^, Watanabe Koichi^1^, Saga Tsuyoshi^1^, Iwanaga Joe^1,2^, Yamaki Koh-ichi^1^

##### ^1^Division of clinical and Gross Anatomy, Department of Anatomy, Kurume University School of Medicine, Kurume, Fukuoka; ^2^ Seattle Science Foundation, Seattle, USA

Introduction: The palmar carpal ligament (PCL) is called the flexor retinaculum; it is a bi-laminar structure comprising the transverse carpal ligament. The flexor retinaculum is the antebrachial fascia (ABF) on the palmar surface of the wrist. The palmar branch of the ulnar nerve runs through the antebrachial fascia and is distributed to the skin near the hypothenar eminence. The purpose of this study was to clarify the morphological structure of the PCL with a focus on the layers of the ABF.

Materials and Methods: Observation and measurements of the hand and PCL were performed in thirteen hands and forearms obtained from seven preserved cadavers. Two different methods were used; one method was normal dissection and the other, cross-section of the tissue.

Results: The ABF on the palmar surface of the wrist was found to form a compartment that covers the flexor carpi radialis, palmaris longus, and flexor carpi ulnaris muscles, as well as the ulnar artery and nerve. In addition, the length of the palmar branch of the ulnar nerve penetrating through the ABF was 5.3 ± 1.6 cm from the pisiform. A statistically significant (P < 0.05) correlation (r = 0.845) was observed length from pisiform to the point which the nerve piercing the ABF and the PCL length.

Conclusion: The layer structure in the palmar surface of the ABF varies according to the location. We believe that surgeons operating in this region should take the details of these anatomic structures into account.

### The innervation of the tensor of the vastus intermedius

#### Takamura Keiko^1,2^, Saiki Kazunobu^1^, Okamoto Keishi^1^, Tsurumoto Toshiyuki^1,2^

##### ^1^Department of the Macroscopic Anatomy, Nagasaki University, Nagasaki; ^2^ Cadaver Surgical Training Center, Nagasaki University, Nagasaki

Introduction: Regarding the quadriceps, Grob (Clin Anat 29:256-263, 2016) reported the existence of a new muscular head between the vastus lateralis (VL) and the vastus intermedius (VI), which was termed, the Tensor of the Vastus Intermedius (TVI). The aims of this study were to investigate the presence and form of the TVI in the Japanese population and to clarify the derivation of the TVI after further nerve analysis of the nerves to the TVI.

Materials and Methods: From June 2018 to July 2019, 36 lower limbs of the 20 Japanese cadavers were investigated. We searched for the TVI, and classified the TVI into the four types of Grob’s classification. In addition, fiber analysis of the nerves innervating the TVI was performed under a stereomicroscope to determine each classification type.

Results: The TVI was identified on each side and the following types were found: independent type, 11%; common type, 29%; VL type, 37%; and VI type, 23%. As a result of nerve fiber analysis, in the common type, the TVI was under dual nerve supply of the nerves from the VL and VI. In the other three types, the TVI was supplied by the nerve from the VL.

Conclusion: The muscular head between the VL and VI seems to be present constantly in Japanese. It was observed that nerves from the VL component were always distributed in this muscular head in the nerve fiber analysis, therefore it is assumed that this muscular head is the most closely related to the VL. It is considered that the name of this muscular head should be “the secondary head of the VL” rather than the TVI.

### Functional anatomical analysis of the human gluteus maximus muscle

#### Anetai Hidaka^1^, Sakai Tatsuo^2^

##### ^1^Department of Anatomy and Life Structure, School of Medicine, Juntendo University, Tokyo; ^2^Department of Physical Therapy, Faculty of Health Science, Juntendo University, Tokyo

Introduction: The human gluteus maximus muscle (GM) is a strong extensor muscle of the hip joint, which transfers the femoral bone dorsally. However, it is generally recognized that most of the GM is inserted into the iliotibial tract. In the present study, we reevaluated the muscle architecture of the GM including its function.

Materials and Methods: From embalmed Japanese cadavers, 50 human GM were collected and dissected by medical students in the gross anatomical practice at the School of Medicine, Juntendo University.

Results: The fascicles of the GM were arranged roughly in parallel, and ran latero-caudally. The superior two-thirds of the GM, “upper main portion”, formed the stout distal tendon. The tendon descended and inserted into the gluteal tuberosity of the femoral bone. Slight fibers of the tendon attached to the femoral bone via the iliotibial tract. The inferior third of the GM, “lower accessory portion”, inserted muscularly to the posterior medial surface of the lateral intermuscular septum of thigh including the distal tendon of the upper main portion.

Conclusion: The human GM was distinguished into two parts, the upper main portion, and the lower accessory portion. The upper main portion of the GM transmits the muscle force to the femoral bone directly and strongly.

## Plenary lecture 2

### Anatomic analysis of stabilizing structures based on tendon, aponeurosis, and joint capsule; reconsideration of the word “ligament”

#### Nimura Akimoto

##### Department of Functional Joint Anatomy, Tokyo Medical and Dental University, Tokyo

Previously, anatomic studies on the musculoskeleton have been focused on the ligamentous structures in each joint. The word “ligament” has been described to consist of fibers, which are less regularly arranged than those of tendons, and more regularly arranged than those of aponeuroses. In other words, the term “ligament” is not the absolute definition, because the structural borders between the tendons, ligaments, and aponeuroses are not so clear to separate them. Therefore, the conventional anatomical methods based on the specific ligaments have their limits in the analysis of anatomical structures in relation to joint stability. Thus, I lectured about the anatomical observations based on the periarticular surroundings, including the tendinous structures and joint capsule. In the elbow and hip joints, I explained the details of the macroscopic findings of the tendinous structures, bony morphology using micro CT, and the histological features of the attachments, different from the “ligaments”.

